# Fabrication of Superhydrophobic Silicone Rubber with Periodic Micro/Nano-Suction Cup Structure by ArF Excimer Laser-Induced Photodissociation

**DOI:** 10.3390/nano9060870

**Published:** 2019-06-07

**Authors:** Masayuki Okoshi

**Affiliations:** Department of Electrical and Electronic Engineering, National Defense Academy, Yokosuka, Kanagawa 239-8686, Japan; okoshi@nda.ac.jp

**Keywords:** silicone rubber, superhydrophobic property, micro/nano-suction cup structure, ArF excimer laser, photodissociation

## Abstract

A 193-nm ArF excimer laser was used to induce the photodissociation of Si–O bonds of silicone rubber in order to fabricate a periodic micro/nano-suction cup silicone structure, approximately 1 μm in diameter and 2 μm in height at regular intervals of 2.5 μm. The laser was focused on Al-coated silicone rubber by each silica glass microsphere 2.5 μm in diameter, which covered the entire surface of the silicone rubber. The silicone rubber underneath each microsphere photochemically swelled after laser-ablating the coated Al to limit the diameter of the swelling. Simultaneously, the coated Al was able to adjust the focal point to the surface of the silicone rubber to form a hole approximately 500 nm in diameter, centered at the swollen silicone. The dependences of the thickness of the coated-Al and the laser pulse number are discussed, based on the observations of a scanning electron microscope (SEM) and an atomic force microscope (AFM). The superhydrophobic property of the fabricated micro/nano-suction cup structure was successfully found.

## 1. Introduction

In many polymers, silicone ([SiO(CH_3_)_2_]_n_) is chemically stable and has useful properties such as heat resistance, cold resistance, and chemical resistance. This polymer also exhibits a high optical transparency and high electrical insulation properties. Therefore, silicone is widely employed for scientific and engineering applications [1,2,3]. Moreover, the polymer has good peeling-resistant and self-adhesive properties that can be applied to industrial and biomedical uses [4,5]. It is also of interest to its variety of uses that there are three forms of silicone: resin, rubber, and oil. On the basis of its various useful properties, the use of silicone is more widespread if the silicone obtains a superhydrophobic property.

The superhydrophobic property, which repels water remarkably, is used to functionalize materials. A silicone or fluorocarbon polymer has a hydrophobic surface because these polymers are composed of CH_3_ or CF_3_ groups, respectively. However, they are still required to have a higher hydrophobicity [6,7]. On the other hand, the superhydrophobic property of a plant leaf or insect exists by nature [8,9]. This property is physically produced by a convex–concave surface structure. Thus, the formation of a convex–concave structure is effective for the realization of a highly hydrophobic material. For instance, the micro/nanostructuring of materials by laser ablation is a popular processing method for producing the superhydrophobic property [10,11,12,13]. Increasing the roughness through filling the micro- and nanofillers within the polymeric matrix was recently reported to also produce hydrophobic properties [14,15].

Thus, to obtain a superhydrophobic property in silicone rubber, we used the basis of our previous photochemical processing of silicone rubber to fabricate the microstructure on the surface. When an ArF excimer laser of 193 nm wavelength irradiates the surface of the silicone rubber, the main chain of Si–O bonds of silicone undergoes photodissociation into smaller molecules, resulting in the swelling of the laser-irradiated area [16,17,18,19,20]. For the micro/nanostructuring, microspheres made of silica glass, 2.5 μm in diameter, were used [21,22]. Very recently, we also found that the subsequent formation of a textured nanometer-sized Al thin film resulted in the addition of a nanostructuring effect to the microstructured silicone. Therefore, a highly superhydrophobic property was successfully obtained [23,24].

In this paper, in order to change the shape of the swollen silicone to a micro/nano-suction cup structure, maintaining the superhydrophobic property, we deposited Al thin film on the silicone rubber before the microsphere alignment and before the laser irradiation. When the aluminum was deposited, the morphology of the thin film was to be textured only on the silicone rubber [23,24]. Thus, the focal point of the ArF excimer laser for each microsphere could be adjusted to the surface of the silicone rubber underneath the Al thin film to form a hole centered at the swollen silicone. Also, before focusing the laser on the surface of the silicone rubber, the Al thin film was ablated to form circular, micron-sized apertures, which can function as a contact mask to limit the diameter of the swollen silicones. Synthetic suction cups are useful for the production of climbing robots and tapes, for instance, on the basis of biomimetics [25,26]. In this paper, we discuss the dependencies of the coated Al thickness and laser pulse number in the fabrication of a periodic micro/nano-suction cup structure in silicone and the simultaneous realization of superhydrophobic properties in the material.

## 2. Experimental Procedure

Aluminum thin film was first deposited on 2-mm-thick silicone rubber by the vacuum evaporation of Al wire (99.99% purity). The silicone rubber was stable during the evaporation. The thickness of the Al thin film was changed from approximately 10 to 100 nm. As measuring the thickness of the silicon rubber with a stylus profilometer (Veeco-Bruker Nano, Dektak^3^, Tucson, AZ, USA) was difficult, a glass slide was used as a substrate for the measurement. Silica glass microspheres approximately 2.5 μm in diameter (Nippon Shokubai, KE-P250, Chiyoda, Tokyo, Japan) were dispersed in ethanol. The dispersed solution was dripped on the surface of the Al-coated silicone rubber. Thus, a single layer of microspheres was formed on the Al-coated silicone rubber, in addition to the removal of excess microspheres [21,22].

The ArF excimer laser (Coherent, COMPexPro110, Santa Clara, CA, USA) irradiated the sample surface without any optics. The beam path was purged by N_2_ gas at a flow rate of 10 L/min to avoid the strong optical absorption of oxygen molecules in the air. The single-pulse fluence of the ArF excimer laser was approximately 10 mJ/cm^2^. The pulse repetition rate was 1 Hz. The laser pulse number was varied from 1 to 1800. The pulse width of the ArF excimer laser was approximately 20 ns. All laser irradiations were carried out at room temperature. After the laser irradiation, the silica glass microspheres were removed by a 1 wt % HF chemical etching and subsequent ultrasonic washing in ethanol [21].

## 3. Results and Discussion

**Figure 1:**
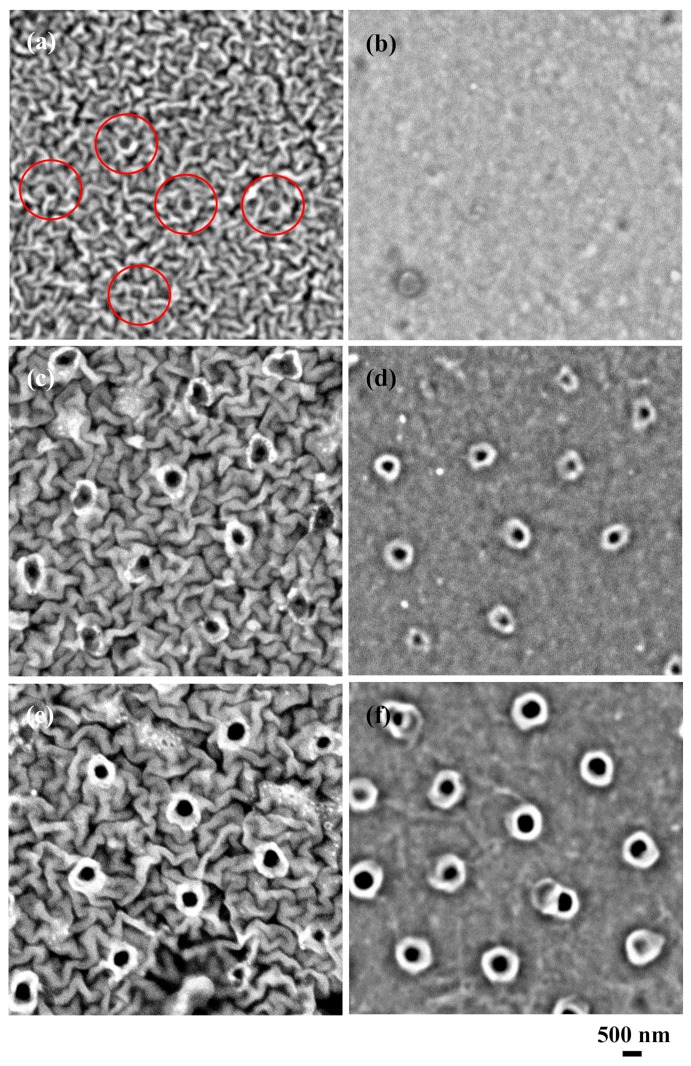
Scanning electron microscope (SEM) images of the 10-nm-thick aluminum-coated silicone rubber after ArF excimer laser irradiation and after the removal of silica glass microspheres. Laser pulse number was varied between (**a**,**b**) 1, (**c**,**d**) 5, and (**e**,**f**) 30 pulses. The left-hand side is before KOH chemical etching and the right-hand side is after KOH chemical etching.

Figure 1 shows the scanning electron microscopy (SEM, Phenomworld, Pro, Waltham, MA, USA) images of the 10-nm-thick Al-coated silicone rubber after ArF excimer laser irradiation and after the removal of silica glass microspheres. In the SEM observations, the working distance was 2 mm and the acceleration voltage was set at 10 kV. The laser pulse number was varied from 1 to 30. In the case of one pulse, several submicron holes were already seen (Figure 1a). In order to see how the laser-irradiated areas of the silicone rubber changed, the sample was immersed in a 0.5 wt % KOH aqueous solution for 30 min to remove the coated Al thin film, as shown in Figure 1b. No holes were found on the surface of the silicone rubber. Thus, in this case, only the Al thin film was ablated by focusing the ArF excimer laser with each microsphere. When the laser pulse number increased to five pulses, in Figure 1c, the laser-irradiated areas underneath each microsphere changed. Circular areas approximately 700 nm in diameter in white were seen, together with circles approximately 300 nm in diameter in black. After the KOH chemical etching, in Figure 1d, the laser-irradiated areas did not change after removing the coated Al thin film.

Thus, we found that the coated Al thin film underneath each microsphere was ablated by focusing the laser with microspheres. Also, the ablated areas of Al thin film functioned as circular apertures approximately 700 nm in diameter to limit the irradiation areas on the silicone rubber. In Figure 1e,f at 30 pulses, the circular areas in white were expanded to be approximately 900 nm in diameter, together with circles approximately 400 nm in diameter in black.

**Figure 2:**
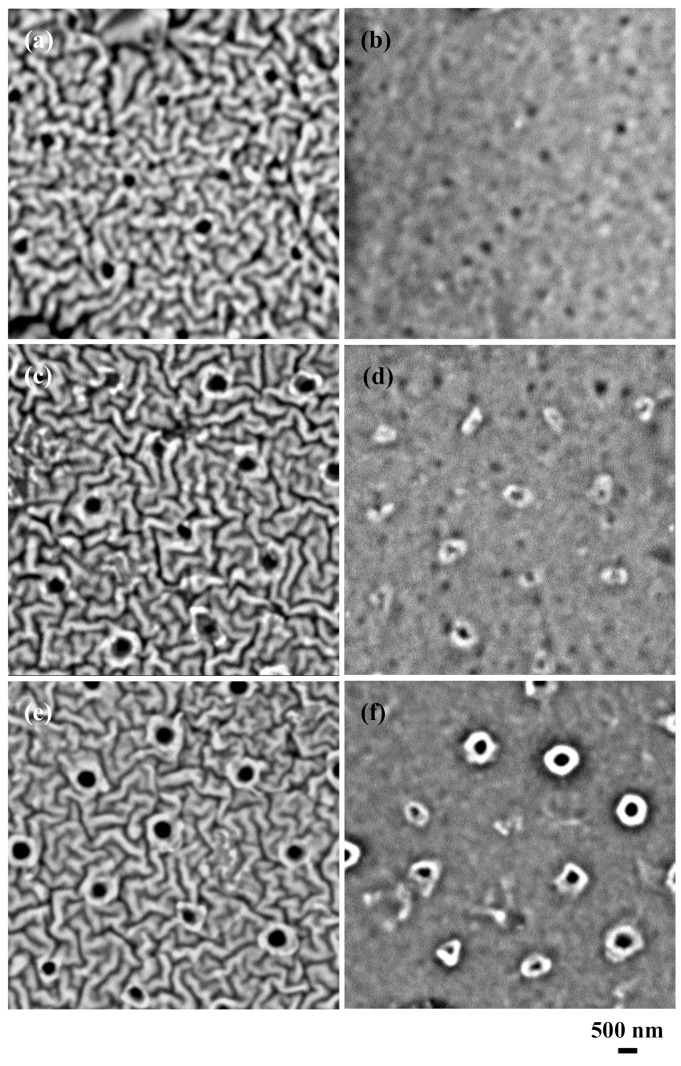
Scanning electron microscope (SEM) images of the 50-nm-thick aluminum-coated silicone rubber after ArF excimer laser irradiation and the removal of silica glass microspheres. Laser pulse number was varied between (**a**,**b**) 1, (**c**,**d**) 5, and (**e**,**f**) 30 pulses. The left-hand side is before KOH chemical etching and the right-hand side is after KOH chemical etching.

Figure 2 shows the SEM images of the 50nm-thick Al-coated silicone rubber after ArF excimer laser irradiation and after the removal of silica glass microspheres. When the laser pulse number was one, in Figure 2a, some submicron-sized holes were recognized on the Al-coated silicone rubber. The morphological changes were not seen on the silicone rubber underneath the Al thin film after KOH chemical etching (Figure 2b). At five pulses, in Figure 2c,d 400-nm-diameter holes in black were seen on the Al-coated silicone rubber. However, the diameter of the laser-irradiated areas was smaller on the silicone rubber. This means that the diameter of the Al holes became smaller at the interface between the Al and the silicone rubber because the Al thin film was thicker. The holes had a tapered shape. As shown in Figure 2e,f in the case of 30 pulses, the diameter of the Al holes was almost identical to that of the laser-irradiated areas on the silicone rubber, although the diameter of the laser-irradiated areas became irregular.

**Figure 3:**
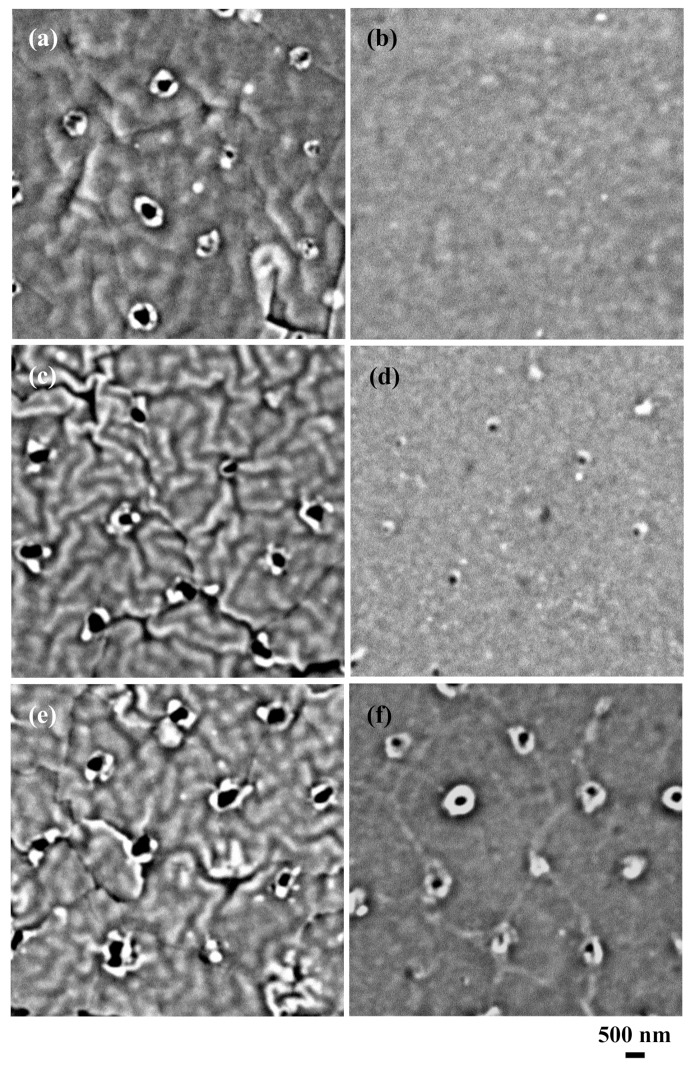
Scanning electron microscope (SEM) images of the 100-nm-thick aluminum-coated silicone rubber after the ArF excimer laser irradiation and after the removal of silica glass microspheres. Laser pulse number was varied between (**a**,**b**) 1, (**c**,**d**) 5, and (**e**,**f**) 30 pulses. The left-hand side is before KOH chemical etching and the right-hand side is after KOH chemical etching.

Figure 3 shows the SEM images of the 100-nm-thick Al-coated silicone rubber after the ArF excimer laser irradiation and after the removal of silica glass microspheres. In the case of one pulse, in Figure 3a, some submicron-sized holes were seen on the Al-coated silicone rubber. On the other hand, the laser-irradiated areas remained unchanged on the silicone rubber after KOH chemical etching (Figure 3b). When the laser pulse number was five (Figure 3c,d), the diameter of the laser-irradiated areas was remarkably smaller on the silicone rubber compared to the case seen in Figure 2d. At 30 pulses (Figure 3e,f), the diameter of aluminum holes was almost the same as that of the laser-irradiated areas on the silicone rubber, although the diameter of the laser-irradiated areas became more irregular compared to the case seen in Figure 2f.

**Figure 4:**
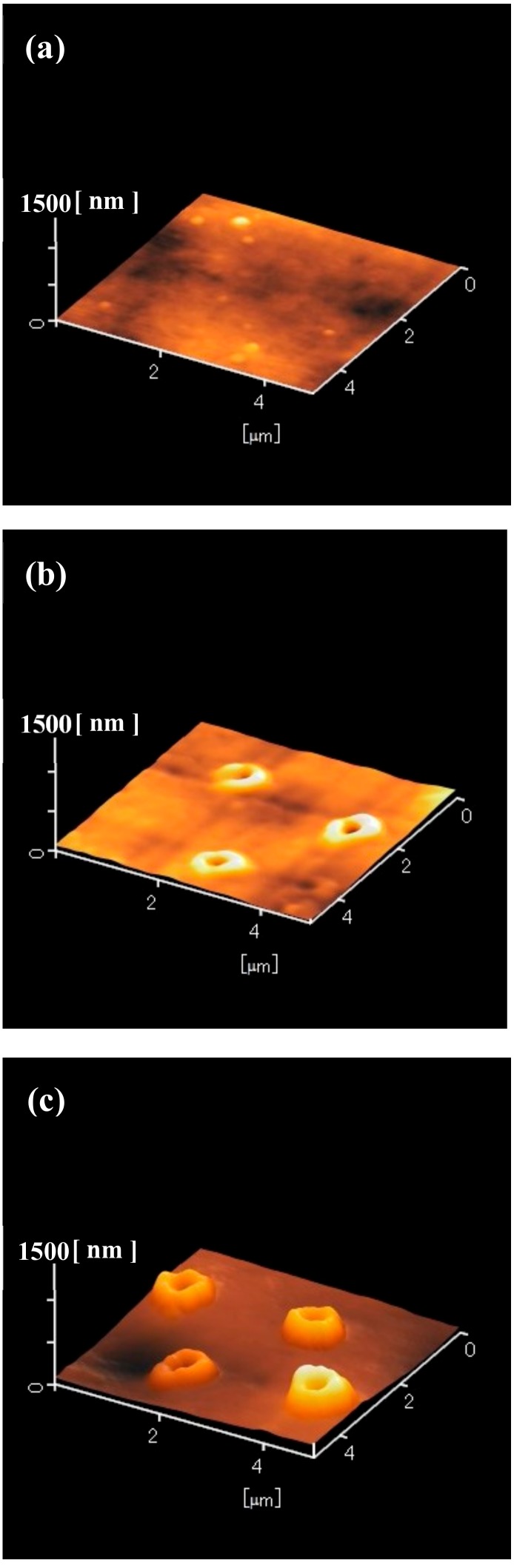
Atomic force microscope (AFM) images of the 10-nm-thick aluminum-coated silicone rubber after the ArF excimer laser irradiation and after the removal of silica glass microspheres. Laser pulse number was varied between (**a**) 1, (**b**) 5, and (**c**) 30 pulses.

**Figure 5:**
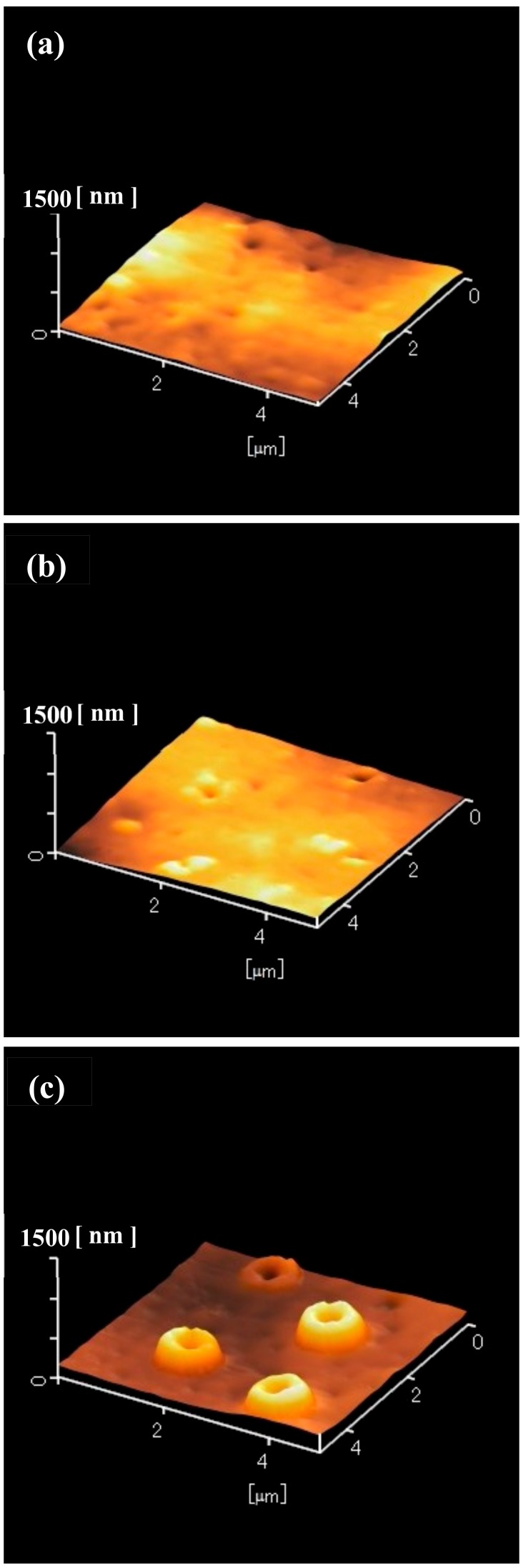
Atomic force microscope (AFM) images of the 50 nm-thick aluminum-coated silicone rubber after ArF excimer laser irradiation and after the removal of silica glass microspheres. Laser pulse number was varied between (**a**) 1, (**b**) 5, and (**c**) 30 pulses.

**Figure 6:**
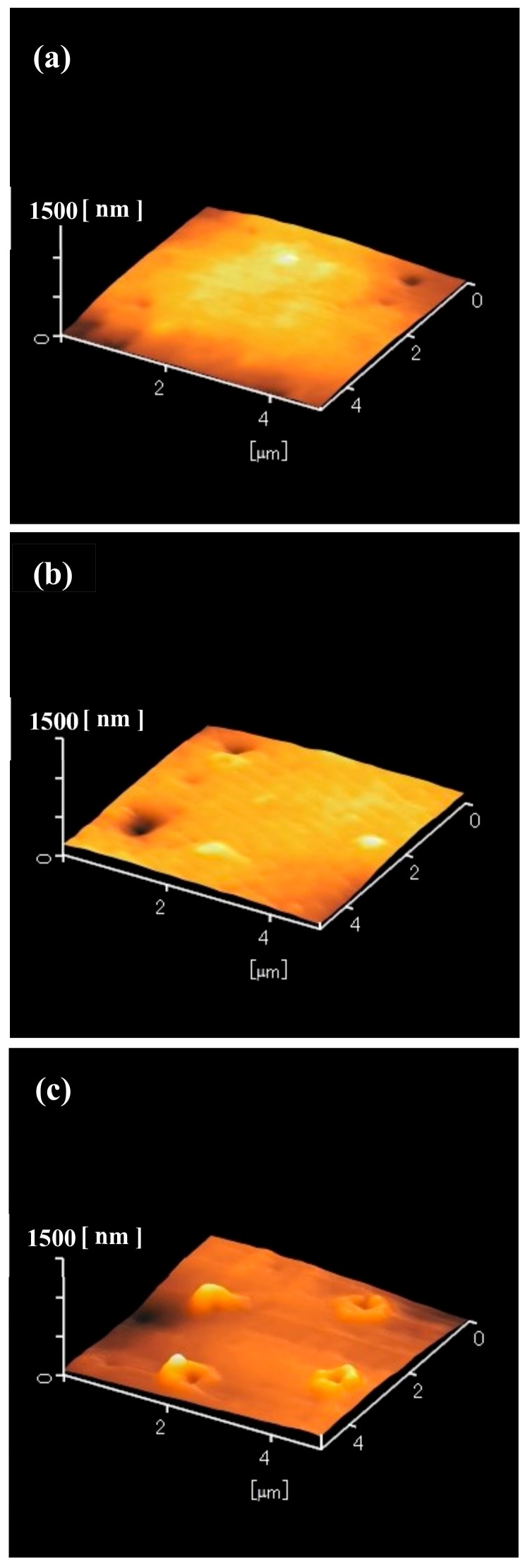
Atomic force microscope (AFM) images of the 100-nm-thick aluminum-coated silicone rubber after the ArF excimer laser irradiation and after the removal of silica glass microspheres. Laser pulse number was varied between (**a**) 1, (**b**) 5, and (**c**) 30 pulses.

To determine the structure of the laser-irradiated areas of the silicone rubber, the samples shown in Figure 1b,d,f were observed by atomic force microscopy (AFM, Hitachi High-Technologies, AFM5100N, Minato, Tokyo, Japan), as shown in Figure 4. The laser-irradiated areas remained unchanged when the laser pulse number was one pulse (Figure 4a). In the case of five pulses (Figure 4b), the laser-irradiated areas swelled. Also, a hole centered at the swollen silicone was observed. At 30 pulses (Figure 4c), the laser-irradiated areas clearly swelled and the height increased. A hole centered at the swollen silicone was also observed. Judging from Figure 1f and Figure 4c, the diameter of the holes was approximately 300–400 nm. However, the depth of the holes could not be measured. The samples shown in Figure 2b,d,f were also observed by AFM, as shown in Figure 5. At one pulse, the laser-irradiated areas were unchanged (Figure 5a). In the case of five pulses, only a tiny hole was recognized (Figure 5b). When the laser pulse number was 30, the laser-irradiated areas clearly swelled. A hole centered at the swollen silicone was also observed. The samples shown in Figure 3b,d,f were also observed by the AFM, as shown in Figure 6. At one and five pulses (Figure 6a,b), the laser-irradiated areas almost remained unchanged. Even in the case of 30 pulses, swelling and holes were only slightly observed.

In our previous work, when the microspheres were directly aligned on the silicone rubber, the holes were not formed on the swollen silicone by laser ablation [21,23]. The focal point of the ArF excimer laser through a microsphere is estimated to be 650 nm below the surface of the silicone rubber. Thus, the silicone rubber was not ablated [21]. On the other hand, in the case of depositing Al thin film onto silicone rubber, the surface morphology of the Al thin film did not become flat, but showed a textured structure [23,24]. Then, the surface roughness was approximately 34 nm, under the condition that the thickness of the Al thin film was 10 nm on a glass slide [24]. Qualitatively, the focal point of the ArF excimer laser could be shifted to the surface side, resulting in exceedance of the ablation threshold of approximately 50 mJ/cm^2^ at a 193 nm wavelength [17]. In addition, debris of the ablated Al might also push up the microsphere to move the focal point closer to the surface. Therefore, the swollen silicone might have a hole approximately 300–400 nm in diameter.

**Figure 7:**
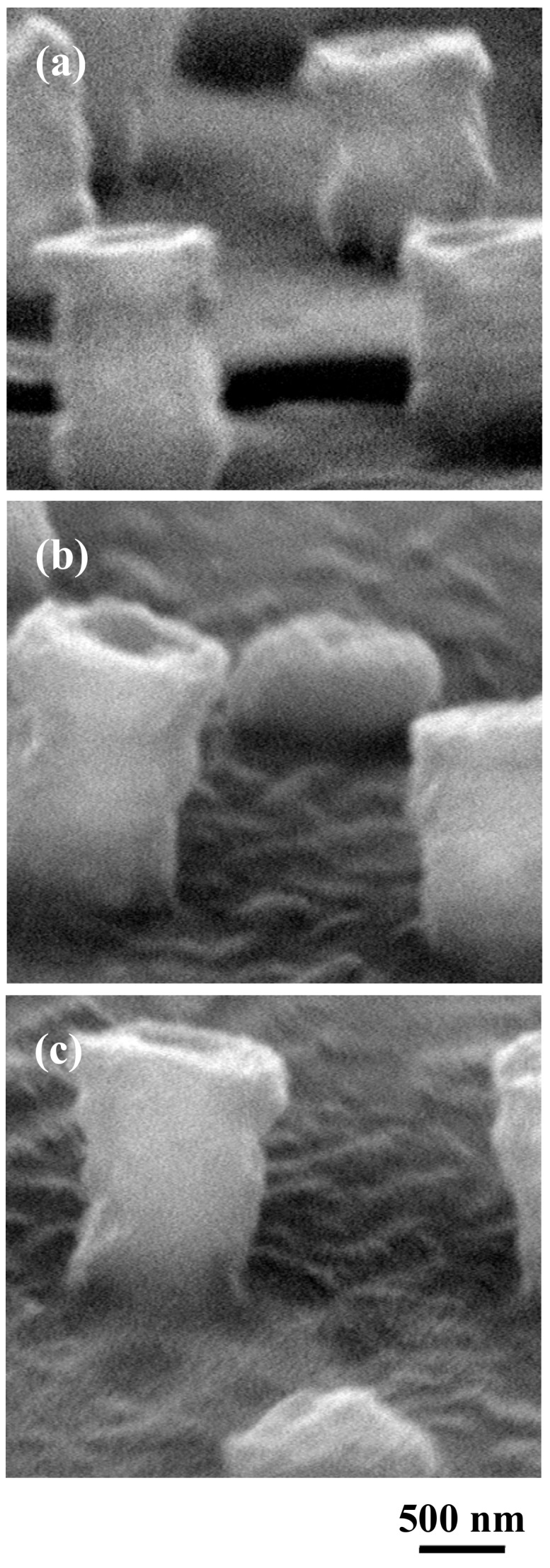
Scanning electron microscope (SEM) images of the laser-irradiated aluminum-coated silicone rubber after the removal of silica glass microspheres. Thickness of aluminum thin film was varied between (**a**) 10, (**b**) 50, and (**c**) 100 nm.

Figure 7 shows the SEM images of the samples after the ArF excimer laser irradiation and after the removal of microspheres. The laser pulse number was 1800. In the case of 10-nm-thick Al thin film, a cylindrical structure approximately 1 μm in diameter and 2 μm in height was clearly observed at regular intervals of approximately 2.5 μm. When the thickness of the Al thin film increased from 10 to 50 and 100 nm, the formation of the cylindrical structure became irregular. Therefore, it seems that an Al coating thickness of 10 nm on the silicone rubber is best for fabricating the cylindrical structure with a hole.

In our previous work, the chemical bonding state of the swollen silicone was analyzed by X-ray photoelectron spectroscopy. The Si 2p signal at 102.1 eV was measured from the swollen silicone, which means that it was a silicone peak [21]. Thus, the property of the fabricated cylindrical structure of the swollen silicone might be almost kept as a silicone rubber, which can be expected to function as a micro/nano-suction cup [27].

**Figure 8:**
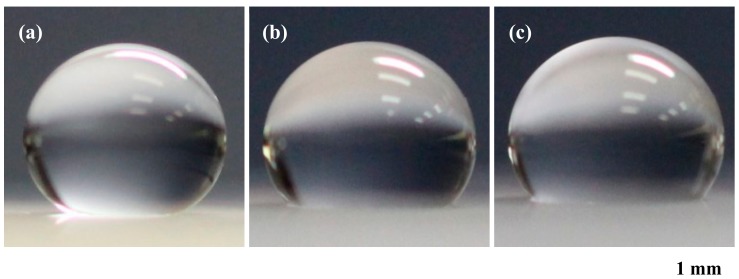
Photographs of water drops on the periodic structure of the swollen silicone with (**a**) 10, (**b**) 50, and (**c**) 100 nm-thick aluminum thin films for measuring the contact angle of water.

To evaluate the hydrophobic property of the samples, the water contact angle was measured [21,23,24], as shown in Figure 8. In the measurement, a drop of water was 10 μL, which was observed from the side by a digital camera. Static contact angles were measured from the photographs. In the case of a bare silicone rubber, the contact angle of water was approximately 90° [21,23,24]. On the other hand, the periodic cylindrical structure of the swelled silicone with 10-nm-thick Al thin film showed a contact angle of approximately 155°, which indicates a clear superhydrophobic property (Figure 8a). When the thickness of the Al thin film increased to 50 and 100 nm, the contact angle of water decreased to 140° and 138°, respectively. This was due to the irregular height of the swollen silicone. To find each optimum condition for the thicknesses of 10, 50, and 100 nm, the laser pulse number was varied from 300 to 900 and 3600. In the case of 50 nm, the contact angles were 135°, 139°, and 142°, respectively. When the thickness was 100 nm, the contact angles were 132°, 135°, and 139°, respectively. In both cases, a superhydrophobic property was not obtained. However, in the case of 10 nm, the contact angles were 141°, 150°, and 156°, respectively. Thus, 900 pulses and over are required to obtain the superhydrophobic property under the condition of a 10-nm-thick Al thin film. Based on these successful results, we will develop a silicone rubber-based microdevice that is capable of holding a small object by each micro-suction cup structure, even in water or other solution [27].

## 4. Conclusions

We used an ArF excimer laser of 193 nm wavelength to induce the photodissociation of the Si–O bonds of silicone rubber to swell the laser-irradiated areas. The laser was focused on the Al-coated silicone rubber by each microsphere made of silica glass, 2.5 μm in diameter, which covered the entire surface of the silicone rubber. The silicone rubber underneath each microsphere photochemically swelled due to the photodissociation, after laser ablation of the coated Al to limit the diameter of the swelling. At the same time, the coated Al enabled the adjustment of the focal point on the surface of the silicone rubber to form a hole approximately 500 nm in diameter centered at the swollen silicone. Thus, periodic micro/nano-suction cup structuring of silicone with approximately 1 μm diameter and 2 μm height at regular intervals of 2.5 μm was successfully achieved. The dependencies of the Al coating thickness and laser pulse number were discussed to see how the laser-irradiated area swelled, as observed by SEM and AFM. The superhydrophobic property of the fabricated micro/nano-suction cup structure was found by measuring the contact angle of water, indicating that the present method can produce superhydrophobic silicone rubber. The present results provide an advanced technique for holding small, nanometer-sized objects with each micro/nano-suction cup structure in water, towards the fabrication of a silicone-based microdevice moving in water for electronic and biomedical applications.
